# Proximate composition, phytochemical analysis, and in vitro antioxidant potentials of extracts of *Annona muricata* (Soursop)

**DOI:** 10.1002/fsn3.498

**Published:** 2017-06-29

**Authors:** Kingsley C. Agu, Paulinus N. Okolie

**Affiliations:** ^1^ Department of Medical Biochemistry School of Basic Medical Sciences College of Medical Sciences University of Benin Benin City Edo Nigeria; ^2^ Department of Biochemistry Faculty of Life Sciences University of Benin Benin city Nigeria

**Keywords:** *Annona muricata*, Antioxidant abilities, Phytochemicals, Proximate compositions

## Abstract

Numerous bioactive compounds and phytochemicals have been reported to be present *Annona muricata* (Soursop). Some of these chemical compounds have been linked to the ethnomedicinal properties of the plant and its antioxidant properties. The aim of this study was to assess the proximate composition, phytochemical constituents and in vitro antioxidant properties of *A. muricata* using standard biochemical procedures. The defatted *Annona muricata* crude methanolic extracts of the different parts of the plant were used for the estimation of proximate composition and phytochemical screening. The crude methanolic extracts of the different parts of the plant were also fractionated using solvent–solvent partitioning. Petroleum ether, chloroform, ethyl acetate, methanol, and methanol‐water (90:10) were the solvents used for the fractionation. The different fractions obtained were then used to perform in vitro antioxidant analyses including, 1, 1‐diphenyl‐2‐picrylhydrazyl (DPPH) radical scavenging ability, ferric reducing properties, and hydroxyl radical scavenging ability. The leaf methanolic extract had a higher lipid content, whereas its chloroform fraction demonstrated a better ability to quench DPPH free radical. The root‐bark methanol‐water, leaf methanol, fruit pulp chloroform, and leaf petroleum ether fractions demonstrated potent ferric reducing properties. The leaf and stem‐bark petroleum ether fractions demonstrated better hydroxyl‐free radical scavenging abilities. The leaf and fruit pulp of *Annona muricata* have a very potent antioxidant ability compared to the other parts of the plant. This can be associated with the rich phytochemicals and other phytoconstituents like phenols, flavonoids, alkaloids, and essential lipids, etc. Significant correlations were observed between the antioxidant status and phytochemicals present. These results thus suggest that some of the reported ethnomedicinal properties of this plant could be due to its antioxidant potentials.

## 1 INTRODUCTION

*Annona muricata* has been attributed numerous health benefits most of which have been linked to its antioxidant potentials (Agu, Okolie, Eze, Anionye, & Falodun, 2017; Agu, Okolie, Falodun, et al., 2017; Ahalya, Shankar, & Kiranmayi, 2014; Baskar, Rajeswari, & Kumar, 2007; Chao‐Ming et al., 1998; Gupta, Pandey, Shah, Yadav, & Seth, 2011; Okolie, Agu, & Eze, 2013; Rupprecht, Hui, & Mc Laughlin, 1990). The antioxidant properties of the plant have been attributed to some of its phytochemicals and phytoconstituents (Agu, Okolie, Eze, et al., 2017; Agu, Okolie, Falodun, et al., 2017; Baskar et al., 2007; Gupta et al., 2011; Okolie et al., 2013; Vit, Santiago y, & Pérez‐Pérez, 2014). Thus, this formed the basis on which this research was designed and carried out. This is to establish the link between the antioxidant status of *Annona muricata* and its phytochemical constituents. *Annona muricata* (Annonaceae) commonly called Soursop due to the soured and acidic nature of the matured and ripe fruit pulp is a small, upright evergreen tree growing 5–10 meters in height. It is a shrubby plant located majorly in the rain forest regions of Nigeria, where it is used locally for several ethnomedicinal purposes—as a laxative and purgative, wound healing, etc. The health benefits of this plant have been attributed to their unique phytochemical composition (Agu, Okolie, Eze, et al., 2017; Agu, Okolie, Falodun, et al., 2017; Okolie et al., 2013; Okwu & Omodamiro, 2005; ; Vit et al., 2014). Many bioactive compounds and phytochemicals, majorly the annonaceous acetogenins and essential oils, have been isolated and elucidated from *A. muricata* (Agu, Okolie, Falodun, et al., 2017; Gleye, Laurensa, Laprevoteb, Seranib, & Hocquemiller, 1999) and its many uses in natural medicine have been validated by scientific researches (Weniger et al., 1986). Intensive chemical investigations of the leaves, fruit pulp, and seeds of different species of this plant have resulted in the isolation of a great number of acetogenins. The phytochemicals present in *Annona muricata* are alkaloids, flavonoids, carbohydrates, cardiac glycosides, saponins, tannins, phytosterols, terpenoids, and proteins (Edeoga, Okwu, & Mbaebie, 2005). Agu, Okolie, Eze, et al. (2017) reported the presence of alkaloids, flavonoids, and phenols in high quantities especially in the fruit pulp and leaf. They also reported the possible hemomodulatory properties of the plant. Some of the isolated compounds from this plant have also displayed some interesting biological and pharmacological activities, such as antitumoral, cytotoxicity, antiparasitic, pesticidal properties (Gleye et al., 1999), etc. These activities have been linked to the antioxidant properties of the plant. Consequently, this research was designed to ascertain the antioxidant properties of *Annona muricata*.

## 2 MATERIALS AND METHODS

### 2.1 Preparation of plant material for proximate and phytochemical analyses

A large quantity of fresh parts of the plant, *i.e*., such as the fruit pulp, leaf, stem‐bark, and root‐bark were collected from trees from household gardens in Benin City and around the University of Benin, Edo state, Nigeria. The plant was identified by Dr Bamidele of the Department of Plant Biology and Biotechnology, University of Benin, and authenticated by Professor Idu of the same department. A voucher specimen number, UBHa 0205, was deposited at the Herbarium of Department of Plant Biology and Biotechnology, University of Benin. The properly washed plant samples were pulverized after drying at room temperature (about 25°C) for 4 weeks, oven‐dried to constant weight and then defatted using Soxhlet extractor. Defatted samples were then used for further proximate analysis. It is instructive to note that this plant under investigation for its health benefits was collected from Ugbowo community in Benin City, Edo state, Nigeria.

### 2.2 Preparation of plant material for phytochemical analyses and in vitro antioxidant assays

The pulverized plant materials were extracted by macerating about 300 g of each parts in 3.8 L of methanol (Jinhuada, JHD, Shantou, Guangdong, China), stirred and left to soak for 72 hr. The mixture was filtered with muslin cloth. To obtain the crude extracts, the filtrates were transferred to a rotary evaporator to separate the solvent from the extract *(in vacuo*). The evaporated extracts were then transferred into airtight containers and stored in a refrigerator at about 4°C until it was required for subsequent phytochemical and in vitro antioxidant analyses. However, before in vitro antioxidant analyses were carried out, the crude methanolic extracts of the different parts of the plant were subjected to solvent‐solvent partition extraction using graded solvent polarity—petroleum ether, chloroform, ethyl acetate, methanol, and methanol‐water—to obtain fractions of the different parts of the plant.

### 2.3 Proximate composition

Proximate contents of the various parts of *Annona muricata* were determined using the methods of A.O.A.C. (2000), gross energy values (GEV) were calculated using the method of Livesey (1990), and caloric values (CV) were estimated using the methods of Ooi, Iqbal, and Ismail (2012) and Codex Alimentrius (1991).

### 2.4 Phytochemical screening

The following phytochemicals were tested for their presence in the plant—tannins (Trease & Evans, 1989), flavonoids (Harborne, 1973), saponins (Evans, 2002), phlobatannins (Trease & Evans, 1989), terpenoids (Edeoga et al., 2005), carbohydrates and monosaccharides (Molischs test, Barfoed's test, Benedict's test) (Evans, 2002), cardiac glycosides (Keller–Killain test) (Edeoga et al., 2005), Bial's test (Pentoses) (Edeoga et al., 2005), ketoses (Seliwanoff's test) (Edeoga et al., 2005), starch (Iodine tests), protein/peptide bonds (Biuret Test), arginine (Sakaguchi's Test), cysteine (Lead sulfide test), aromatic amino acids (Xanthoproteic test), phenolic amino acids (Million's test), anthraquinones (Sofowara, 1982), and alkaloids (Trease & Evans, 1989).

### 2.5 In vitro antioxidant evaluation

1, 1‐diphenyl‐2picrylhydrazyl (DPPH) radical scavenging ability was tested, using method described by Singh, Murthy, and Jayaprakasha (2002) and Jain et al. (2008). Ferric reducing or antioxidant powder were determined as described by Oyaizu (1986) and Zhao et al. (2008), respectively. The ability to scavenge hydroxyl radical was measured by the reduction in nitro blue tetrazolium (NBT) according to a previously described method (Hazra, Biswas, & Mandal, 2008).

### 2.6 Statistical analysis

Data were entered into the Microsoft excel spread sheet (version 10) prior to descriptive analysis. The data were represented as mean ± SEM. Correlation analyses were done using the Pearson's correlation of the IBM Corp^®^ Statistical Package for Social Sciences, SPSS^®^, Version 21.0. Histograms and line plots were done using Graph Pad software^®^ Prism 5, Version 5.01 (2007).

## 3 RESULTS

### 3.1 Proximate analyses

**Figure 1:**
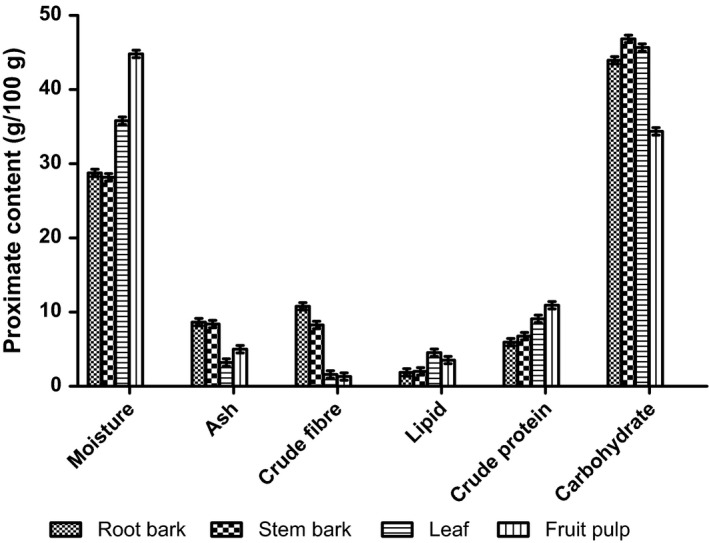
Proximate composition of the various parts of *Annona muricata* analyzed

**Figure 2:**
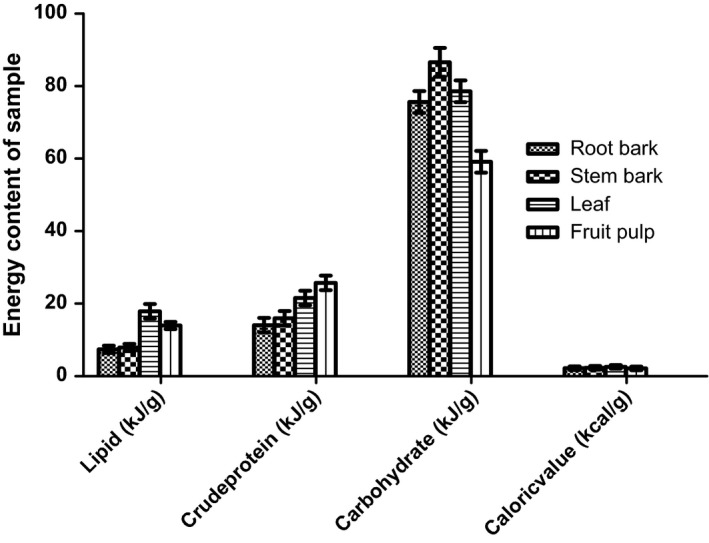
Gross energy values (kilojoules per gram) and caloric values (kilocalories per gram) of the various parts of *Annona muricata*

The analyses of proximate composition of the various parts of *Annona muricata* as represented by Figure 1 (fruit pulp, leaf, stem‐bark, and root‐bark) showed that the fruit had the highest moisture content followed by leaf, whereas the stem‐bark had the lowest moisture content. The root‐bark had the highest ash content followed by the stem‐bark and fruit. Crude fiber was highest for the root, followed by the stem‐bark, and lowest for leaf and fruit. Lipid content was highest in leaf, followed by the fruit, and with the least recorded level for root‐bark. However, the highest level of crude protein was observed for fruit and leaf, whereas root‐bark had the lowest level. Carbohydrate levels were relatively within the same range for root‐bark, leaf, and stem‐bark, with the lowest level recorded for fruit. The high level of moisture in the fruit may be responsible for the comparatively low carbohydrate observed. The caloric values as represented by Figure 2 demonstrated that leaf had the highest (2.60 kcal./g). The caloric values for stem‐bark, root‐bark, and fruit pulp were, 2.32 kcal./g, 2.17 kcal./g, and 2.14 kcal./g, respectively.

### 3.2 Phytochemical analysis of fruit, leaf, stem‐bark, and root‐bark of *Annona muricata*

The results of phytochemical screening of methanolic extracts of various parts of *Annona muricata* is represented in Table 1. All the phytochemicals tested were present, apart from phlobatannins and anthraquinones (George, Kumar, Rajkumar, Suresh, & Kumar, 2012). However, some of these phytochemicals were quantified and reported by Agu, Okolie, Eze, et al. (2017).

**Table 1:** Phytochemical screening of the various parts of *Annona muricata*

Phytochemical	Fruit	Leaf	Root‐bark	Stem‐bark
Tannins	+	+	+	+
Flavonoids	+	+	+	+
Saponins	+	+	+	+
Phlobatannins	–	–	–	–
Terpenoids	+	+	+	+
Carbohydrates	+	+	+	+
Cardiac glycosides	–	+	+	–
Reducing sugars	+	+	+	+
Monosaccharides	+	+	+	+
Pentoses	+	+	+	+
Ketoses	+	+	+	+
Starch	+	+	+	+
Protein	+	+	+	+
Arginine	+	+	+	+
Cysteine	+	+	+	+
Aromatic amino acids	+	+	+	+
Phenolic amino acids	+	+	+	+
Anthraquinones	–	–	–	–
Alkaloids	+	+	+	+
Steroids	+	+	+	+
Phenolics	+	+	+	+
Nitrogen and halides (Cl^−^)	+	+	+	+
Sulfur and sulfate ion	+	+	+	+
Nitrate ion	+	+	+	+

### 3.3 Assessment of in vitro antioxidant capacities of the different parts of *Annona muricata*

**Figure 3:**
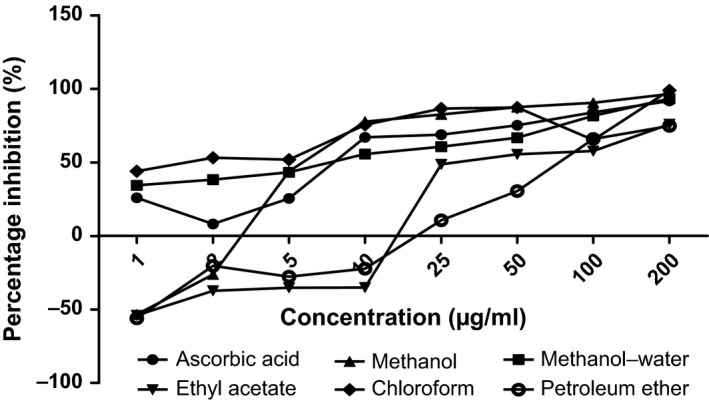
1, 1‐diphenyl‐2‐picrylhydrazyl (DPPH) radical scavenging ability of fruit fractions and ascorbic acid (%)

**Figure 4:**
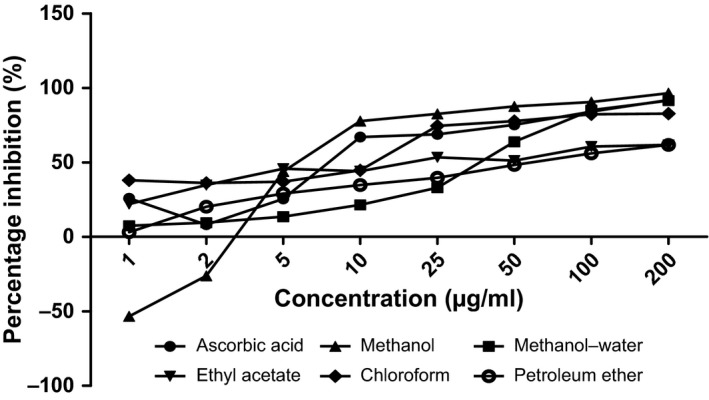
1, 1‐diphenyl‐2‐picrylhydrazyl (DPPH) radical scavenging ability of leaf fractions and ascorbic acid (%)

**Figure 5:**
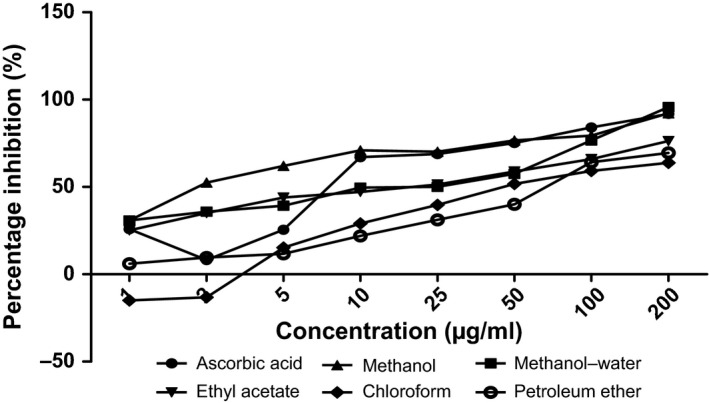
1, 1‐diphenyl‐2‐picrylhydrazyl (DPPH) radical scavenging ability of stem‐bark fractions and ascorbic acid (%)

**Figure 6:**
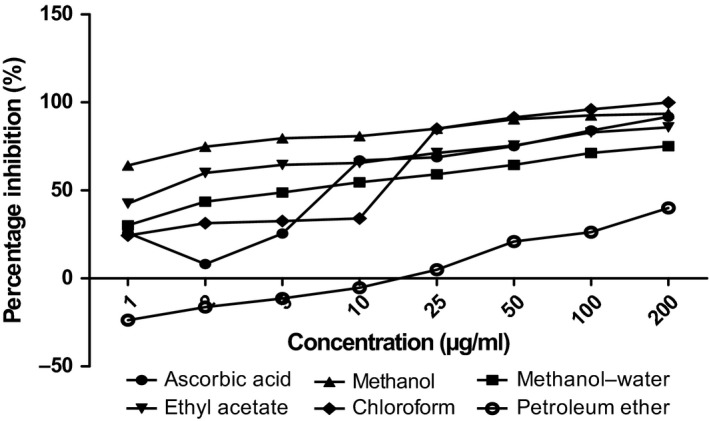
1, 1‐diphenyl‐2‐picrylhydrazyl (DPPH) radical scavenging ability of root‐bark fractions and ascorbic acid (%)

**Figure 7:**
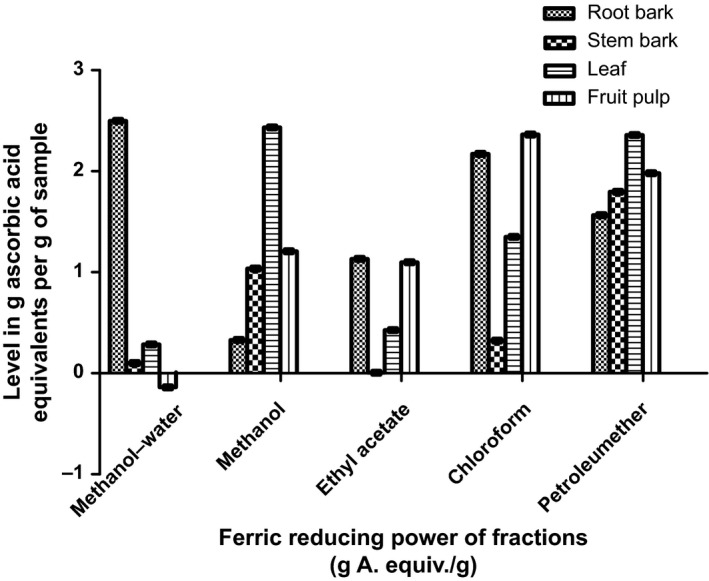
Levels of reducing power of *Annona muricata* fractions

**Figure 8:**
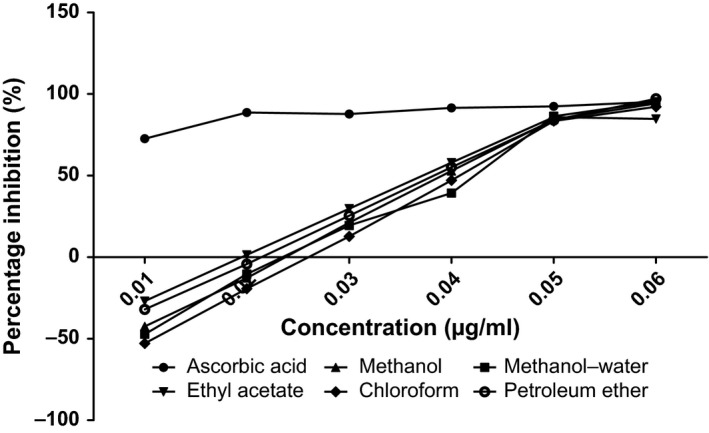
Percentage hydroxyl radical (OH ^•^) radial scavenging ability of ascorbic acid and fruit fractions

**Figure 9:**
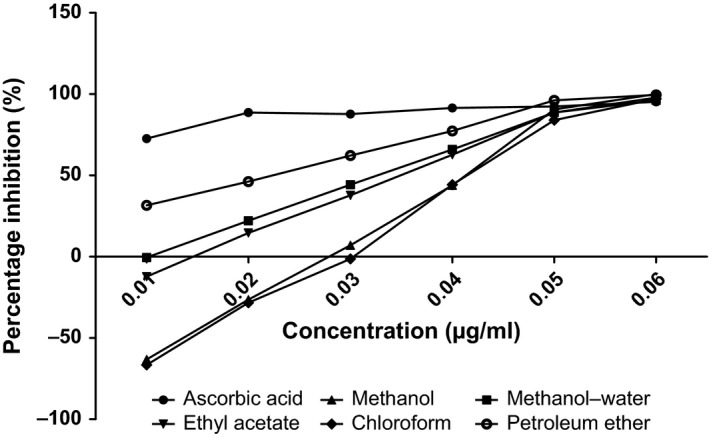
Percentage hydroxyl radical (OH ^•^) radial scavenging ability of ascorbic acid and leaf fractions

**Figure 10:**
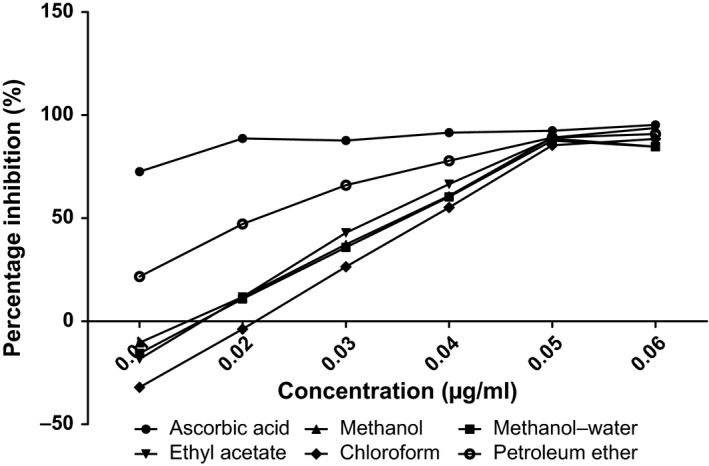
Percentage hydroxyl radical (OH ^•^) radial scavenging ability of ascorbic acid and stem‐bark fractions

**Figure 11:**
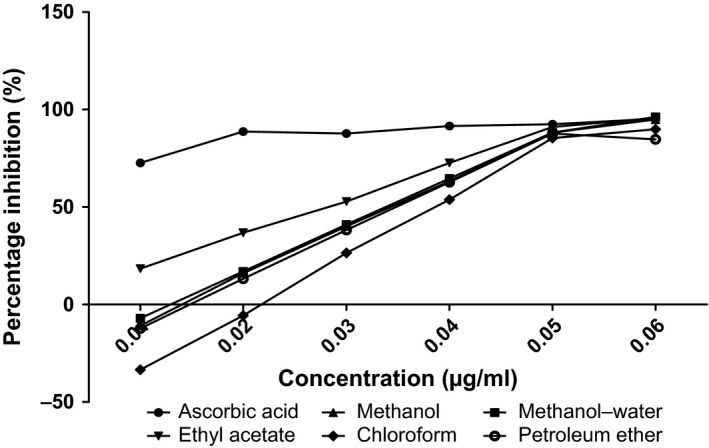
Percentage hydroxyl radical (OH ^•^) radial scavenging ability of ascorbic acid and root‐bark fractions

**Figure 12:**
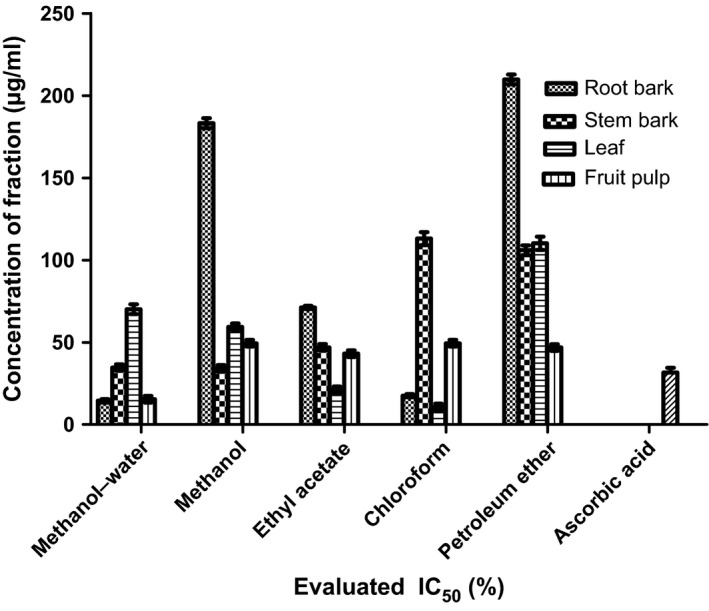
Evaluated IC_50_ of various fractions of *Annona muricata* based on the DPPH radical scavenging ability

**Figure 13:**
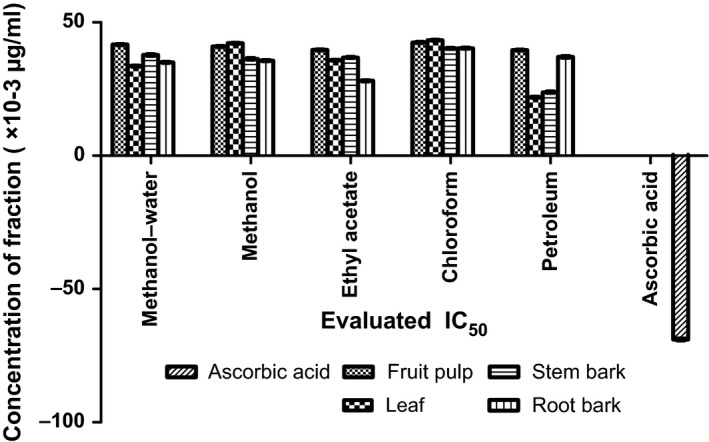
Evaluated IC_50_ of the various fractions of *Annona muricata* based on the hydroxyl radical (OH•) radial scavenging ability

The antioxidant capacities of fractions of the various parts of *Annona muricata* are represented; DPPH scavenging ability (Figures 3, 4, 5, 6), reducing power (Figure 7), and hydroxyl radical scavenging ability (Figures 8, 9, 10, 11). The DPPH and hydroxyl radical scavenging abilities were extrapolated to obtain the 50 % inhibitory concentrations (IC_50_) of the extracts (Figure 12 and Figure 13), respectively. The fruit fractions displayed better abilities to detoxify DPPH radical, compared to other parts of the plant. The leaf chloroform fraction performed better, followed by root‐bark methanol‐water, fruit methanol‐water, and leaf ethyl acetate fraction, in a decreasing order. Figure 12 represents the reducing power of *Annona muricata* fractions—the root‐bark methanol‐water, leaf methanol, fruit chloroform, and leaf petroleum ether fractions demonstrated better reducing powers compared to other fractions. The hydroxyl radical (OH^●^) scavenging abilities of the various fractions showed that the leaf petroleum ether fraction was able to detoxify OH^●^ better than the other fractions. Stem‐bark petroleum ether, root‐bark ethyl acetate, and leaf methanol‐water, also exhibited potent hydroxyl radical quenching abilities.

The antioxidant data obtained were correlated with the phytochemical contents of the fractions (Agu, Okolie, Eze et al., 2017) using the Pearson's correlation (r). Some of the tests were also extrapolated to obtain 50 % inhibitory concentrations (IC_50_). Ascorbic acid which was used as the reference standard for DPPH radical scavenging ability test gave an IC_50_ of 32.6 μg/ml. On a general assessment, the fruit fractions showed better abilities to quench the DPPH radical (ranging from 15.4 μg/ml to 49.4 μg/ml) (Padmini et al., 2014), compared to the other parts of the plant.

But on a fraction‐specific comparison, the leaf chloroform fraction (10.7 μg/ml) performed better, followed by root‐bark methanol‐water (15.4 μg/ml), fruit methanol‐water (15.4 μg/ml), and leaf ethyl acetate fraction (21.1 μg/ml), in decreasing order. These were the only fractions that had IC_50_ lower than that of the reference standard (ascorbic acid). The DPPH scavenging abilities of the fruit and leaf fractions could be attributed to their high total flavonoids and phenol contents.

The hydroxyl radical scavenging ability of the various fractions showed that petroleum ether fraction of the leaf (21.9 × 10^−3 ^μg/ml) (Baskar et al., 2007) performed better than the other fractions of the plant which could be attributed to the alkaloid content with a significant correlation (*r* = .736 and *p* = .023), compared to ascorbic acid reference standard (−68.9 × 10^−3 ^μg/ml). Stem‐bark petroleum ether (23.8 × 10^−3 ^μg/ml), root‐bark ethyl acetate (28.1 × 10^−3 ^μg/ml), and leaf methanol‐water (33.6 × 10^−3 ^μg/ml), also exhibited potent hydroxyl radical detoxifying abilities.

The high hydroxyl radical scavenging ability of the stem‐bark petroleum ether fraction could be linked to the high total flavonoids content (*r* = .782, *p* = .041); root‐bark ethyl acetate to the alkaloid content (*r* = .610, *p* = .03); and leaf methanol‐water to the total flavonoids content (*r* = .426, *p* = .027). The ability of the various plant fractions to reduce oxidized compounds (metals) was also assessed. Root‐bark methanol‐water fraction displayed the most potent oxidant reducing ability (2.50 g.AE/g) (Ahalya et al., 2014). This was followed by leaf methanol (2.44 g.AE/g), fruit chloroform (2.36 g.AE/g), and root‐bark chloroform (2.20 g.AE/g) (Baskar et al., 2007). Fruit methanol‐water fraction showed the least comparative performance of −0.14 g.AE/g.

The ability of the root‐bark methanolic fraction to reduce oxidized compounds showed a significant correlation with its total phenols content (*r* = .892, *p* = .037); the leaf methanolic fraction showed a significant correlation with its alkaloidal content (*r* = .793, *p* = .041); the fruit chloroform fraction showed a significant correlation with its total flavonoids content (*r* = .910, *p* = .039); and the root‐bark chloroform fraction showed a significant correlation with its alkaloidal content (*r* = .629, *p* = .047).

## 4 DISCUSSION

Researchers have pointed out the possible link between the antioxidant potentials of *Annona muricata* and its health benefits. However, the antioxidant properties of *Annona muricata* have been observed to have rich phytoconstituents such as annonaceous acetogenins, essential oils/fatty acids and other phytochemicals including, alkaloids, polyphenols, flavonoids, tannins, etc., (Fang, Rieser, Gu, Zhao, & Mc Laughlin, 1993; Gupta et al., 2011; Rupprecht et al., 1990).

Information about the comparative studies of antioxidant profile of the various parts of the *Annona muricata* is very limited. Thus this formed the basis for this study. The abilities of fractions of *Annona muricata* to mitigate free radical damage, to chelate and reduce metals, and to quench deleterious hydroxyl radicals were assayed as markers of antioxidant potentials. This research was able to identify and established that *Annona muricata* possesses potent antioxidant properties. The observations in this study on the antioxidant potentials of *Annona muricata* could provide a mechanistic explanation of its reported ethnomedicinal importance (Agu, Okolie, Eze, et al., 2017; Agu, Okolie, Falodun, et al., 2017; Ahalya et al., 2014; Fang et al., 1993; Gupta et al., 2011; Okolie et al., 2013; Rupprecht et al., 1990; Vit et al., 2014). It is also expected that the data and findings from this research will provide a base‐line information for researchers who have reported possible links of antioxidant properties of *Annona muricata* with antineoplastic, wound healing, antihyperlipidemic, antiplasmodial, antileishmanial, etc. This could open up further investigations into other health benefits of the plant.

### 4.1 Phytochemical screening and proximate analysis

All the phytochemicals tested for were present except phlobatannins and anthraquinones. This finding confirms the report of George et al. (2012). The proximate composition of the various parts of *Annona muricata* (fruit pulp, leaf, stem‐bark, and root‐bark) showed that the fruit has the highest moisture content followed by leaf. The stem‐bark has the lowest moisture content. The root‐bark has the highest ash content followed by the stem‐bark and fruit in decreasing order. The moisture and ash contents of the fruit and root‐bark, respectively, were expected. This is due to the high fluid content of the fruit pulp and the proximity of the root to the soil as source of mineral elements. Capillary action of the transporting vessels of the plant against gravity could also be a major contributory effect to the transport of minerals from the root, through the stems and branches, and then to the storage sites, that is, the leaf and fruit for metabolic usage.

Crude fiber content was highest in root. This was followed by the stem‐bark, leaf and fruit, in decreasing order. The root and the stem of the plant play among other functions, the major role of transport needed nutrients for plant metabolism. Thus, it houses a very large network of woody and fibrous transport vessels (xylem and phloem). The fruit and leaf sections of the plant play the major roles of general plant metabolism, storage of metabolites and biomolecules. Consequently, there is less requirement of these woody materials.

Lipid content was highest in leaf. This was followed by fruit and root‐bark, in decreasing order. The presence of high amount of essential oils in the fruit pulp of *Annona muricata* have been reported by Kossouoh, Moudachirou, Adjakidje, Chalchat, and Figuérédo (2007). The high lipid contents in the fruit pulp and leaf corroborates the findings of Kossouoh et al. (2007) considering that *Annona muricata* is very rich in annonaceous acetogenins which are derivatives of long chain fatty acids (C32 or C34) (Chao‐Ming et al., 1998; Gupta et al., 2011; Rupprecht et al., 1990). This partially suggests that *Annona muricata* possesses a high fatty acids and lipid synthetic and storage ability, especially in the fruit. Fekam, Amvam, Menut, Lamaty, and Bessière (1996) and Soheil, Mahmoud, and Rodney (2004) also pointed out that terpenoids, esters of aliphatic acids and volatile oils are the major types of lipids present in the leaf and fruit pulp of *Annona muricata*. However, 10.9 g/100 g and 9.1 g/100 g were the amount of crude protein recorded for fruit and leaf, respectively, and 6.8 g/100 g for stem‐bark. Root‐bark had the least level of crude protein (5.9 g/100 g). The fruit pulp protein content of this research disagrees with an earlier report of 1.0 g/100 g (Chao‐Ming et al., 1998). Carbohydrate levels were relatively within the same range (43.9–46.9 g/100 g) for root‐bark, leaf and stem‐bark, with the lowest level recorded for fruit (34.4 g/100 g). The observed fruit pulp carbohydrate level contradicts the reported level of 16.8 g/100 g by Chao‐Ming et al. (1998). The high level of moisture in the fruit pulp of *Annona muricata* could be responsible for the low carbohydrate compared to other parts.

### 4.2 Antioxidant analysis

Data for the in vitro antioxidant profiles for *Annona muricata* are scarce. However, the data for in vitro antioxidant assays obtained in this study were extrapolated to obtain 50 % inhibitory concentrations (IC_50_) for the various fractions of *Annona muricata*. Ascorbic acid was used as the reference standard with an IC_50_ of 32.6 μg/ml for DPPH scavenging ability. The fruit fractions showed better abilities to quench the DPPH radical (Padmini et al., 2014), compared to the other parts of the plant. The fruit methanol‐water and leaf chloroform fractions had the highest abilities of scavenging DPPH radicals which could be attributed to their high total flavonoids and phenol contents (*p* < .05). The leaf and stem‐bark petroleum ether fractions were able to detoxify hydroxyl radicals better than other fractions *Annona muricata* due to their total flavonoid and alkaloid contents (*p* < .05). In terms of the ability of *Annona muricata* to chelate and prevent oxidation by metals, root‐bark methanol and leaf methanol demonstrated better abilities due to their total phenol and alkaloid contents (*p* < .05). These findings agree with the reports of Baskar et al. (2007), Ahalya et al. (2014), Gavamukulya, Abou‐Elella, Wamunyokoli, and El‐Shemy (2014), Gordillo, Ortiz, Larrahondo, and Pachón (2012) and Bryan‐Thomas (2016). Significant correlations (*r*,* p* < .05) were observed between some of the phytochemicals present in *Annona muricata* (Agu, Okolie, Eze, et al., 2017) and its antioxidant status (Vit et al., 2014).

The fruit and leaf fractions of *Annona muricata* demonstrated better abilities to ward off oxidative challenges as described above, compared to the fractions of other parts of the plant. These abilities apart from being associated with their good phytochemicals contents (Baskar et al., 2007; Gavamukulya et al., 2014), could also be linked to their high essential oils (lipid) contents as has been previously reported by Kossouoh et al. (2007), Fekam et al. (1996) and Soheil et al. (2004).

## ACKNOWLEDGMENTS

The effort of Dr. Kingston O. Onyijen of the Department of English and Literature, University of Benin, in making sure the English constructions of this research article is perfected is highly appreciated. We also appreciate the contributions of Professor Abiodun Falodun, Professor A. Obi (Department of Biochemistry, University of Benin), Dr. Ighodaro Igbe, Mrs. Florence Adesina (Department of Pharmacology and Pharmacognosy), Pharm. Dr. Osayenmwere Erharuyi and Mrs. Obi (Department of Pharmaceutical Chemistry). Also, Dr. John Chukudi Anionye, Dr. Stephen Oghagbon and Dr. Richard Edosa (Department of Medical Biochemistry), Mr. Faith Omorogbe, Mr. Jeremiah Mohammed, Mr. Osiregbemhe, Mr. Christopher Ugbodaga, Mr. Reuben Ofeimun, Ms. Courage Ukhuoya, Mr. and Mrs. Jeremiah Agu are all acknowledged for their kind assistance during this research.

## CONFLICTS OF INTEREST

None declared.
